# Effects of Culture Conditions on the Performance of *Arthrospira platensis* and Its Production of Exopolysaccharides

**DOI:** 10.3390/foods11142020

**Published:** 2022-07-08

**Authors:** Zihan Li, Yuhuan Liu, Ting Zhou, Leipeng Cao, Yihui Cai, Yunpu Wang, Xian Cui, Hongbin Yan, Roger Ruan, Qi Zhang

**Affiliations:** 1 State Key Laboratory of Food Science and Technology, Engineering Research Center for Biomass Conversion, Ministry of Education, College of Food Science and Technology, Nanchang University, Nanchang 330047, China; 352313320026@email.ncu.edu.cn (Z.L.); liuyuhuan@ncu.edu.cn (Y.L.); 091442@ncu.edu.cn (L.C.); caiyihui@email.ncu.edu.cn (Y.C.); wangyunpu@ncu.edu.cn (Y.W.); cuixian@ncu.edu.cn (X.C.); 7901118031@email.ncu.edu.cn (H.Y.); 2 Centre for Technology in Water and Wastewater, School of Civil and Environmental Engineering, University of Technology Sydney, Ultimo, NSW 2007, Australia; ting.zhou-3@student.uts.edu.au; 3 Center for Biorefining, Department of Bioproducts and Biosystems Engineering and Department of Food Science and Nutrition, University of Minnesota, Saint Paul, MN 55108, USA; ruanx001@umn.edu

**Keywords:** *Arthrospira platensis*, medium recycle, nitrogen source, exopolysaccharides, growth inhibition

## Abstract

Exopolysaccharides (EPS) produced by *Arthrospira platensis* (*A. platensis*) has been widely applied in industry and commerce for its various activities but the accumulation of EPS in culture medium may influence the growth of *A. platensis* reversely. This work aims to explore the impacts of initial pH, nitrogen source and concentration, phosphate concentration and recycle times of the culture medium on the growth of *A. platensis* and the secretion of its EPS. The results showed that EPS accumulated with the increase in recycle times of culture medium. The optimal initial pH for the growth of *A. platensis* was 8.50, and high pH of 11.5 inhibited the growth of biomass while resulting in highest EPS content of 92.87 mg/g DW. Excessive and limited nitrogen (NaNO_3_ of 25.00 g/L and NaNO_3_ < 2.50 g/L) and phosphate (K_2_HPO_4_ of 5.00 g/L and K_2_HPO_4_ < 0.50 g/L) inhibited the biomass production of *A. platensis* by 1.28–30.77% and 14.29–45.05%, respectively. EPS yield of 97.57 mg/g DW and 40.90 mg/g DW were obtained under NaNO_3_ of 25.00 g/L and K_2_HPO_4_ of 5.00 g/L due to salt stress. These findings are beneficial in providing a theoretical basis for high yield EPS from *A. platensis* without affecting biomass yield.

## 1. Introduction

Microalgae are attracting growing interest in the field of high-value products production, including food, nutraceuticals, cosmetics, and pharmaceuticals [1]. Specifically, *Spirulina* (*Arthrospira platensis*) products are widely promoted as “superfoods” due to their high contents in protein, essential amino acids, vitamins, and minerals [2]. 

Nowadays, the operational costs of cultivating microalgae, notably the costs of the culture media and the regulation of inhibitors in the medium, are the main barriers for scaling-up this bioprocess [3,4]. Large amounts of water are used during microalgae cultivation, and this negatively impacts the economic viability and the environmental sustainability of the process [5]. The overall cost of Zarrouk culture medium is not sustainable in the long run as the approximate cost per liter of culture medium is USD 0.08, which accounts for about 35% of the total cost of biomass production [6]. Recycling of the culture medium after biomass harvesting can reduce the water demands and nutrients by 84% and 55%, respectively, which is essential to reduce the water footprint and energy consumption of *Spirulina* production [7]. To address the challenges associated with large-scale outdoor microalgae cultivation such as low biomass productivity and increased costs, semi-continuous cultivation systems outperformed the conventional batch and/or continuous systems. Hence, semi-continuous cultivation is the mode widely applied for the outdoor cultivation of *Spirulina* [8].

However, the secondary metabolites, non-consumed nutrients and cellular debris accumulate in the recycled medium [9,10]. These cause several changes in physical properties of the recycled medium, such as the increase in alkalinity, the uncertain concentration of nutrients, the contamination of other microorganisms, the debris of microalgae, and the dissolved organic matters (DOM), may pose negative effects on the next cycle of culture [11]. 

Cyanobacterial commonly secrete the secondary metabolites of dissolved organic carbon matters (DOM) into the surrounding environment during growth, especially produced as a protection against suboptimal culture conditions. Those DOM are mainly composed of exopolysaccharides (EPS), saccharide-protein, free fatty acids and so on [12]. EPS are defined as exopolysaccharides that can be excreted by microalgae in the environment around them or be linked to the cell walls of these microorganisms. The functions of EPS include protecting the microalgae cells from biotic and abiotic stresses, mainly dehydration or toxic substances [13].

Compared with the fresh medium, the growth rate of microalgae cultured in the recycled medium decreased with the increase in the number of recycle time of the medium [14], and the accumulated EPS may influence the rheological properties of the culture medium [15]. Moreover, the decomposition of the organic matter results in the increase in bacterial population; in particular, ciliates seem to thrive well under these conditions [10]. EPS are macromolecules with highly diverse chemical compositions and structures, which related to species and growth conditions [16]. Filali Mouhim et al. found that the EPS production is clearly coupled to growth [17]. While Mariana concluded that the EPS production was not associated with the growth rate of microalgae [18]. Obviously, a better understanding of the medium condition on the growth and EPS release of microalgae will no doubt help minimize nutrient cost and/or increase productivities of biomass and EPS in cultures grown outdoors [19]. 

Besides, it was reported that the EPS have high potential as exploitable resources in food, cosmetic and pharmaceutical production processes for its function of antibacterial, antioxidant and anti-inflammatory properties after purification [20]. With intrinsic viscosities, comparable or higher than those of commercial thickening agents, exopolysaccharides of cyanobacteria could be a promising source of novel food hydrocolloids [21]. The polysaccharides (PS) isolated from *Spirulina* (*Arthrospira* sp.) EPS exhibited a non-Newtonian shear-thinning nature, a predominant gel-like behavior and a good resistance to consecutive heating-cooling cycles. The property shows the potential use of EPS in several food processing operations such as mixing, stirring, pumping, and swallowing of food products [15]. 

In addition, there are some other limitations in *Spirulina* semi-continuous cultivation. One of the major limitations is the uncertain changes in nutrients content which may affect the growth and metabolism of *Spirulina* [2]. It was reported that cultures in open ponds under arid and semiarid climates, daily evaporation amounts to 1–2 cm of the water level, leading to a progressive increase in the salt concentration in the culture, and the cells are thus subjected to salinity stress [10]. However, *Spirulina* can proliferate and consume a large amount of nutrients under the suitable environment, which result in the inadequate nutrition in the recycled culture medium [22]. Detailed knowledge of the nutrient uptake kinetics of *Spirulina* in large-scale open pond systems or in the high-pH and high-oxygen medium is lacking.

It is important to note that high metabolites produced under stressed conditions are usually correlated with low biomass productivity during the cultivation of *Spirulina* [15]. The pH [23], nutrients [24], and growth conditions [9] not only play significant roles in the metabolic activities and biomass production of microalgae but also affect the secretion of EPS during the cultivation. 

To the best of our knowledge, the effect of initial pH, nutrient source and concentration on the productions of biomass and EPS by *A. platensis* have not been fully investigated. Nitrogen and phosphate limitation were known as stressed conditions, which favor the accumulation of some reserve products and the production of EPS from cyanobacteria. However, the influence of excessive nitrogen and phosphate on the production of EPS has rarely been reported. Ammonium or urea is commonly used as a cheap alternative nitrogen source of nitrate for *Spirulina* in the industry, but there are also few studies that have reported its impact on the EPS secretion. In addition, high pH levels which have been observed in nutrient-enriched marine systems may have an inhibitory effect on phytoplankton metabolism via the carbonate system, but very little was conducted to study the biochemistry of the major metabolic activities [10,25]. Previous studies investigated the effect of pH on microalgae growth, pigment production, and protein content of *Spirulina.* The direct effect of initial pH value on the cultivation of *Spirulina* and especially its EPS production requires further elucidation. 

Therefore, to address this knowledge gap, the effects of the reuse times, initial pH, nitrate concentration, ammonium concentration, phosphate concentration on the growth and EPS production of *A. platensis* were studied in this work. In this study, the effect of the culture condition on *A. platensis* was quantified by the measurement of biomass and EPS production. Moreover, the mechanism of these abiotic factors affecting EPS secretion was explored to provide a theoretical basis for obtaining high-yield EPS without affecting the biomass production of *A. platensis*. Furthermore, according to the stimulating effect of different culture conditions on EPS secretion, the physical and chemical properties of the recycled medium can be adjusted according to the output requirements, so as to improve the production efficiency and the utilization rate of nutrient resources.

## 2. Materials and Methods

### 2.1. Microalgae and Culture Medium

*Arthrospira platensis* (FACHB: GY-D18) was purchased from the Institute of Hydrobiology Chinese Academy of Science, PR China. Deionized water was used in all cases. The cell suspension of *A. platensis* was first cultivated in 300 mL modified Zarrouk’s medium at 32 °C for 7 days. After centrifuged at 5000 rpm for 5 min, the cultures were seeded into 500 mL Erlenmeyer flasks under continuous illumination of 1200 Lux in modified Zarrouk’s medium at 32 °C, according to Zhou et al. [26]. All of the Erlenmeyer flasks and the medium were sterilized at 121 °C for 20 min previously.

### 2.2. Experimental Design

To investigate the effects of medium recycling on the growth of *A. platensis* and the accumulation of EPS in the recycled medium, the microalgae-free medium harvested after 10 days of culture was used as the recycled medium (RM) and cycled for three times, respectively. The fresh Zarrouk standard medium (FM) was used as the control to observe the effects of recycle times on the growth inhibition and EPS secretion of *A. platensis*.

**Figure 1:**
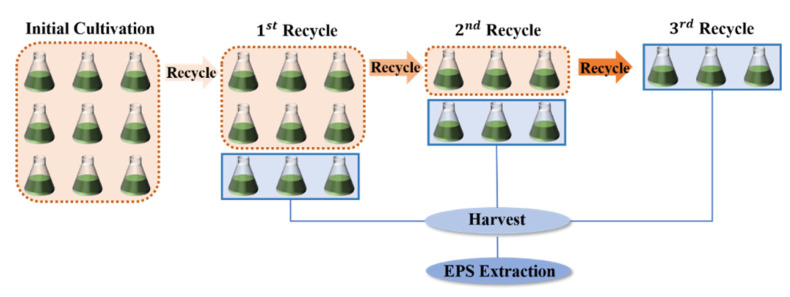
Experimental design of the medium recycle.

To ensure the volume of each cycle was consistent, *A. platensis* required for the three-cycle culture was prepared in the initial culture. The culture time, 10 days, was used in our study because it mimics the real-world *A. platensis* cultivation process in local plants and the maximum EPS production of *A. platensis* was obtained. The experimental design is shown in Figure 1. The details of the culture conditions are described in Section 2.1.

To investigate the effect of nitrogen sources and concentration on the growth of *A. platensis* and the secretion of its EPS, various nitrogen sources and concentrations of culture medium were established and listed in Table 1. The details of the culture conditions are described in Section 2.1. 

To investigate the effect of phosphate concentration on the growth of *A. platensis* and the secretion of its EPS, K_2_HPO_4_ at different concentrations of 0.00, 0.10, 0.50, 1.00 and 5.00 g/L (0.00, 22.79, 113.97, 227.90, and 1139.70 P mg/L) were established. Moreover, 0.50 g/L of K_2_HPO_4_ and 2.50 g/L of NaNO_3_ were the standard phosphate and nitrogen concentration in the Zarrouk medium, and it was used as control. The details of the culture conditions are described in Section 2.1.

To determine the effect of initial pH on the growth of *A. platensis* and the secretion of its EPS, the initial pH of Zarrouk medium was adjusted to 5.5, 7.0, 8.5, 10.0 and 11.5 by adding acid (0.1 M HCl) or alkali (0.1 M NaOH). The details of the culture conditions are described in Section 2.1.

All of the experiments were carried out independently in triplicates.

### 2.3. Microalgae Growth Analysis

The dry cell weight (*DCW*) was calculated according to the method of Zhou et al. [26]. An aliquot of 20 mL suspension of *A. platensis* was filtered by the oven-dried and pre-weighted Whatman filter paper (0.45 μm) before drying at 105 °C for 24 h. The *DCW* and biomass production (*BP*) were defined as follows:

(1) $$DCW=\left(W_{P+M}-W_{P}\right)/0.02$$

(2) $$BP=DCW_{harvest}-DCW_{initial}$$

where *W_P+M_* is the total dry weight (g) of the filter paper and microalgae, and *W_P_* is the dry weight (g) of the filter paper. *DCW_harvest_* is the dry cell weight of the end of the experiment and *DCW_initial_* is the dry cell weight of the beginning of the experiment. The growth rate (*μ*) was determined by Equation (3):

(3) $$µ=\left(lnx_{2}-lnx_{1}\right)/\left(t_{2}-t_{1}\right)$$

where *x_1_* and *x_2_* are the biomass production at time *t*_1_ and *t*_2_, respectively [27].

Growth inhibition (*GI*) of *A. platensis* was defined according to the following equation:

(4) $$GI=\left[\left(DCW_{\mathrm{control}}-DCW_{\mathrm{sample}}\right)/DCW_{\mathrm{control}}\right]\times 100\%$$

where *DCW_control_* and *DCW_sample_* are the dry cell weight (g) of the control and the treatment, respectively.

### 2.4. Culture Medium Analysis

The daily pH of the medium was measured with a Shanghai Lemag pH meter (PHS-3G, INESA Scientific Instrument Co., PR China). The culture medium was taken from each treatment and centrifuged at 10,000 rpm for 10 min at 25 °C. The supernatant was filtered through Whatman filters (0.45 μm). 

Then, the filtrate was concentrated to its 1/10 volume by rotary evaporation at 55 °C. Thereafter, the contained EPS was precipitated by ice cold ethanol (4 volumes of alcohol for one volume of concentrated filtrate) at 4 °C for 12 h [20]. Afterwards, the mixture was centrifugated at 10,000 rpm for 10 min. The pellets were dissolved in 20 mL of ultra-pure water, and placed in a bag (Solarbio, Beijing, China) with a molecular-weight-cut-off (MWCO) of 8–10 kDa and dialysis for 72 h in deionized water to eliminate residual salt [28]. Finally, the samples were freeze-dried and weighted.

### 2.5. Statistical Analysis

All of the experiments were conducted in triplicates. Data plotting were performed with Microsoft Excel 2013, Origin 2017, IBM SPSS Statistics 26, and GraphPad Prism 5.0. Analysis of variance (ANOVA) was carried out wherever applicable and *p <* 0.01 was regarded as a significant difference. For all of the figures and tables, data were presented as mean ± standard deviation (n = 3) of the three independent replicates.

## 3. Results and Discussion

### 3.1. Effects of Medium Recycle on the Growth and EPS Secretion of A. platensis

**Figure 2:**
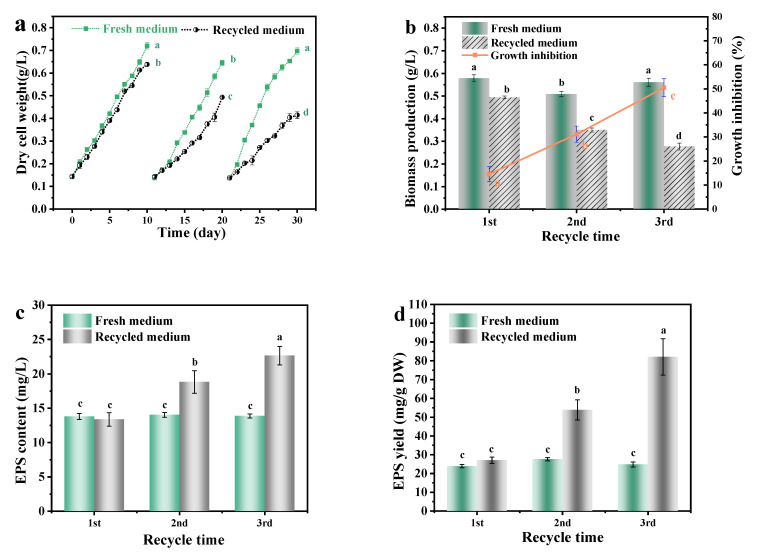
The effects of medium recycling on the growth and EPS secretion of *A. platensis*: (**a**) Growth curves; (**b**) Biomass production and growth inhibition; (**c**) EPS content; (**d**) EPS yield; DW: dry weight; a–d: Different letters above the bars indicated a significant difference among the groups. Points showed average of three independent measurements; vertical bars indicated the standard deviation. (*p <* 0.01, n = 3).

At the end of the culture, *A. platensis* production with the fresh medium was significantly (*p* < 0.01) higher than that in recycled medium (Figure 2). With the increase of medium recycle time, the inhibition of the recycled medium on growth and biomass production of *A. platensis* was gradually increased. Biomass production of *A. platensis* that cultured in the 1st, 2nd, 3rd recycled medium were 85.50%, 68.90% and 52.10% of the fresh medium, respectively. 

As can be seen from Figure 2c, with the increase in medium recycle times of the culture medium, the concentration of EPS and the inhibition that *A. platensis* suffered both significantly increased (*p <* 0.01). After the third recycle time, the EPS content in the recovered medium was 1.70 times that of the initially recovered medium. Such results were consistent with the findings of Depraetere et al. [9]. During the culture process, microalgae could excrete large amounts of dissolved organic matter (DOM) and accumulated in the culture medium. The DOM mainly consisted of EPS. EPS could cause an increase in the viscosity of the medium and inhibit the growth of *A. platensis*, through the reduction in the quantum yield of photosystem II and the change in the biomass composition [14]. This might mainly contribute to the decreased biomass production of *A. platensis* as well. 

As it mentioned, the EPS after purification has a high potential as an exploitable resource in food, cosmetic and pharmaceutical production. In terms of the continuous accumulation of EPS in recycled medium, the directional extraction of high value-added EPS from the recycling process is promising. However, the composition, amount and functions of EPS obtained from microalgae vary according to factors, such as strains and growth conditions. Thus, the biotechnological steps for the large-scale microalgae EPS production must be optimized [13]. Additionally, due to the different compositions and structures of EPS obtained under different culture conditions, the differences in their functional activities and microalgal inhibition mechanisms need to be further explored.

### 3.2. Effects of Initial pH on the Growth and EPS Secretion of A. platensis

pH is one of the most critical environmental conditions in microalgae culture systems. Most *Spirulina* require an optimal pH value of approximately 9.0–9.5. Maintaining a pH of over 9.5 is mandatory in *Spirulina* cultures in order to avoid contamination of other microorganisms [10,23,25]. The variation of pH altered the equilibrium between CO_2_ and HCO_3_^−^ in the medium and change the availability of nutrients for microalgae and transform the photosynthetic mechanisms of microalgae [29].

**Figure 3:**
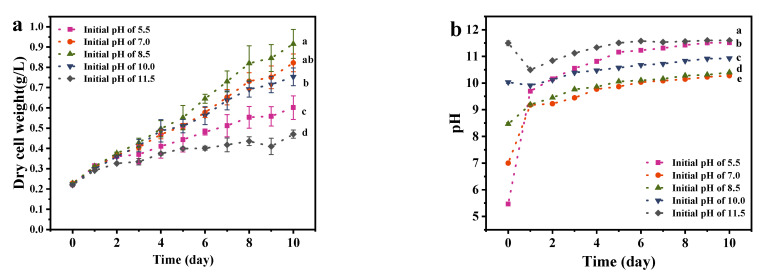

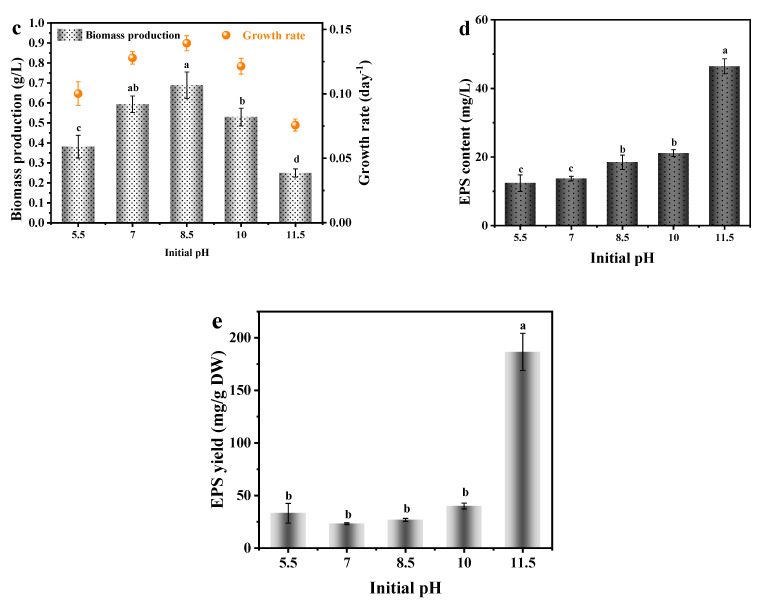
The effects of initial pH medium on the growth and EPS secretion of *A. platensis*: (**a**) Growth curves; (**b**) pH value; (**c**) Biomass production and growth rate; (**d**) EPS content; (**e**) EPS yield; DW: dry weight; a–e: Different letters above the bars indicated a significant difference among the groups. Points showed average of three independent measurements; vertical bars indicated the standard deviation. (*p* < 0.01, n = 3).

As shown in Figure 3, *A. platensis* could grow continuously in the medium with a different initial pH value *A. platensis* reached the maximum biomass production with initial pH of 8.5, followed by the pH value of 7, 10, 5.5 and 11.5. Ogbonda et al. [23] also found that *Spirulina* sp. obtain the highest biomass at pH of 8.5. This could be attributed to the optimal capacities of both photosynthesis and respiration which are associated with its enzymatic activities within the associated pH. Xu et al. [30] found an ATPase associated with the plasma membrane fraction of *Spirulina* reached optimal activity under pH value of 8.5.

As is shown in Figure 3, pH could increase to 9.7 within 24 h and keep accumulate biomass continuously with the initial pH of 5.5. In fact, the photosynthesis of microalgae consumed the inorganic carbon sources in the medium, changed the carbonate equilibrium system and increased the pH [31]. However, biomass production and cell growth rate of *A. platensis* cultured at an initial pH of 5.5 were significantly (*p <* 0.01) lower than that of initial pH of 7.0, 8.5 and 10.0. Since the medium initial pH of 5.5 was adjusted by adding acid, in that case, the carbon dioxide would be released from medium during adjustment, which lead to the reduction in available carbon sources in the medium and further decreased biomass production and EPS secretion.

Though *A. platensis* is an alkalophilic organism, the initial pH of 11.5 poses a strong inhibition on the biomass production of *A. platensis*, which was consistent with the result found by Shi et al. [32]. Higher pH limited the availability of CO_2_ for *A. platensis*, this further inhibited the growth of cells [25]. At an initial pH of 11.5, *A. platensis* flocs appeared and aggregated at the bottom of the flasks on day 2. Cells began to decompose on day 4, accompanied by the obvious precipitation of the biomass and the stratification of supernatant, which may be related to the flocculation function of EPS. Aggregation of cells commonly caused the limited light transmission and oxygen permeation, which further threated the growth of *A. platensis.* In the culture of *Spirulina platensis*, it was observed that the photosynthetic activity became very low and the cultures gradually lost their natural color, a murky froth developed and a putrid odor was evident when the pH was close to 12 [33]. Pogoryelov et al. [34] found that the main inhibition of *A. platensis* by pH was located at the acceptor side of PSII, and a key membrane constituent in it, the oxygen evolving complex (OEC), which could not tolerate high pH value. 

As can be observed from Figure 3e, the EPS yield of *A. platensis* cultured at an initial pH of 11.5 was the highest, up to 92.87 mg/g DW, which was 2.52 times that in the optimal growth condition (initial pH of 8.5). Normally, the EPS secretion of microalgae was a self-protection mechanism that against adverse environments [35]. Under a strong alkaline environment (medium initial pH of 11.5), the supply of carbon source in the culture medium is sufficient, but the availability and metabolism of nitrogen may be inhibited. 

The increase in medium pH is one of the bottlenecks of *A. platensis* semi-continuous cultivation. This study first revealed the effect of initial pH on the EPS production of *A. platensis* and found that strong alkaline environment (initial pH of 11.5) inhibited its growth but favored its EPS secretion. This finding provides new idea to optimize the yield of EPS by regulating the initial pH of the culture medium.

### 3.3. Effects of Nitrogen Source and Concentration on the Growth and EPS Secretion of A. platensis 

**Figure 4:**
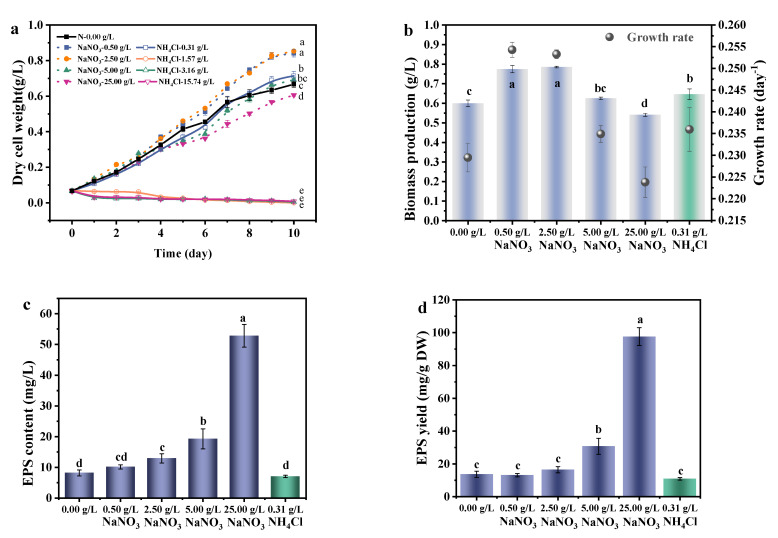
The effects of nitrogen source and concentration on the growth and EPS secretion of *A. platensis*: (**a**) Growth curves; (**b**) Biomass production and growth rate; (**c**) EPS content; (**d**) EPS yield; DW: dry weight; a–e: Different letters above the bars indicated a significant difference among the groups. Points showed average of three independent measurements; vertical bars indicated the standard deviation. (*p <* 0.01, n = 3).

Nitrogen is an important source for the cultivation of *A. platensis*, and it has great influence on microalgae growth, which is dependent upon the amount, the availability, and the type of the nitrogen source [36]. Nitrates usually used as standard nitrogen sources, and ammonium nitrogen used to be a less expensive alternative nitrogen source. As shown in Figure 4, the growth curve and biomass production obtained by cultivation under 0.50 g/L of NaNO_3_ was quite similar to that obtained under 2.50 g/L of NaNO_3_, suggesting that 0.50 g/L of NaNO_3_ seems to be enough to support growth of *A. platensis*. The maximum biomass production of *A. platensis* was obtained under a NaNO_3_ concentration of 2.50 g/L. In contrast, poor biomass production was observed when the initial NaNO_3_ concentration were 0.00 g/L and 25.00 g/L. Though *A. platensis* could fix the nitrogen in the air, the absence of nitrogen nutrient could significantly (*p <* 0.01) decrease the biomass production. Biomass production of *A. platensis* cultured with 5.00 and 25.00 g/L of NaNO_3_ was 0.62 and 0.54 g/L, while biomass production of *A. platensis* cultured with 0.50 and 2.50 g/L of NaNO_3_ was 0.77 and 0.78 g/L, respectively. The NaNO_3_ concentration over 5.00 g/L might pose a salt stress to *A. platensis*. Biomass reduction is often associated with a decrease in photosynthetic activity of microalgae when cultured under conditions of salt stress [19]. Several studies have demonstrated that salt stress would damage the thylakoid membrane of *Spirulina platensis*, decrease oxygen evolution activity and reduce the PSII electron transport by increasing the number of the QB-nonreducing reaction centers [15].

However, *A. platensis* could only grow when the NH_4_Cl concentration was lower than 0.32 g/L. High concentration of NH_4_Cl had significant inhibitory effect on the photosynthetic system, which specifically targeted the oxygen-evolving complex, causing photo-damage of Photosystem II [37]. The main inhibitory form of ammonium is free ammonia which can penetrate into the cells through cell membranes and accumulate in the cell, which is harmful to microalgae [38]. Since *A. platensis* could not grow under the NH_4_Cl concentration of 1.57, 3.16, and 15.74 g/L, the biomass production, growth rate and EPS yield of the corresponding treatments were not measured. 

Figure 4c shows that the EPS production increased with the increase in NaNO_3_ concentration in the culture medium. When the NaNO_3_ concentration increased to 25.00 g/L, the EPS production of *A. platensis* was 52.82 mg/L, which was 4.09 times of that under the nitrogen concentration of 2.50 g/L. As shown in Figure 4b, the growth rate of 0.00 g/L NaNO_3_ was lower than the optimal NaNO_3_ concentration of 2.50 g/L because of the nitrogen limitation. It has been put forward that the synthesis of exocellular polysaccharides in cyanobacteria plays a major role in protecting cells from stress in extreme habitats and other harmful conditions [39]. As mentioned above, the 5.00 and 25.00 g/L NaNO_3_ concentration may be the salt stress to *A. platensis*, and these secreted EPS might play an important role in maintaining the moisture or provides a repository for water, thereby acting as buffers between cells and the atmosphere [40]. The results showed that *A. platensis* cultured with NaNO_3_ produced more EPS and biomass than cultured with NH_4_Cl at the same nitrogen concentration of 0.02 g/L. This finding is consistent with that of Bafana [41] who also found that NaNO_3_ supported greater EPS production as compared to NH_4_Cl. Another possible reason may be that NH_4_Cl could inhibits the nitrate reductase enzyme and therefore affect the biomass production [37].

Most studies focus on the effect of nitrogen limitation on EPS yield of *A. platensis*, and there are few reports related to the effect of excessive nitrogen on EPS yield. Studies have shown that nitrogen limitation may promote the redirection of carbon metabolism toward incorporation into extracellular polymers, induce the over synthesis of exopolysaccharides [42]. While the response of cyanobacteria to nitrogen limitation is not univocal, the responses of cyanobacteria to alterations of culture conditions are strain-dependent [40]. Further, EPS produced by the same species may possess different biotechnology properties [41]. Since the high EPS yield could obtain from the excessive nitrogen culture, the composition, structure, function and application of this kind of EPS deserves further investigate. 

### 3.4. Effects of Phosphate Concentration on the Growth and EPS Secretion of A. platensis

**Figure 5:**
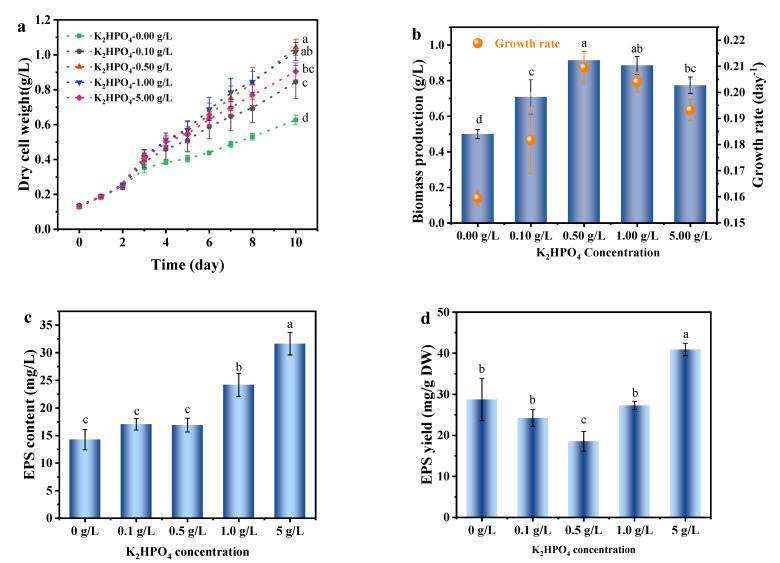
The effects of phosphate concentration on the growth and EPS secretion of *A. platensis*: (**a**) Growth curves; (**b**) Biomass production and growth rate; (**c**) EPS content; (**d**) EPS yield; DW: dry weight; a–d: Different letters above the bars indicated a significant difference among the groups. Points showed average of three independent measurements; vertical bars indicated the standard deviation. (*p <* 0.01, n = 3).

Figure 5 shows the growth rate and biomass production of *A. platensis* reached the highest under phosphate concentration of 0.50 g/L. The lower growth rate and biomass production accompanied with the increase in EPS production at 0.00 and 0.10 g/L of K_2_HPO_4_ might result from the phosphate limitation. This finding was consistent with another cyanobacterium *Cyanothece* 16Som2, of which the exopolysaccharide release increased under conditions of phosphorus-limitation [43]. The excessive phosphate (>0.5 g/L) did not promote the growth of microalgae. In addition, excessive phosphate caused the over-accumulation of polyphosphate in cells and resulted in the destruction of membrane permeability and enzyme function, which might further cause the binding of phosphorus to intracellular components [44].

When the phosphate concentration of medium exceeded 0.50 g/L, the EPS production of *A. platensis* increased with the increase in phosphate concentration, and reaching 31.64 mg/L at phosphate concentration of 5.00 g/L (Figure 5). This is similar to the results of the nitrogen experiment above, that is, salt stress significantly (*p <* 0.01) increased EPS yield. Hiroaki [45] reported that the exopolysaccharides production of *Aphanocapa halophilic*, increased with the increase in phosphate concentration in the culture medium, which was consistent with the results of this study. Vonshak [10] found a significant change in biomass composition of two *Spirulina* strains when grown under salt stress conditions, mainly reflected in the increase in carbohydrates and the decrease in the protein. Accompanying with these changes, EPS could be excreted as a byproduct of overflow metabolism, regulating excessive carbon in cells and balancing intracellular carbon [40]. Thereby the optimization of the culture condition is necessary for the production of high-value compounds in cell or the co-products such as EPS. 

The effect of phosphate concentration in the medium on the growth and EPS secretion of *A. platensis* was similar to the results obtained under other medium conditions, that is, the EPS secretion of *A. platensis* mainly occurred when the cell growth was inhibited. To address the contradictory between biomass growth and EPS production, two-stage cultivation has been attracted the interest that the optimal conditions are provided for maximum biomass production in the first stage, whereas transferred to the stressed conditions which induced the accumulation of target compounds in the second stage [46].

It turns out that the EPS production of *A. platensis* cultured with excessive nitrogen medium (25.00 g/L of NaNO_3_) was 4.09–5.21 times higher than those cultured with nitrogen-limited medium (0–0.5 g/L of NaNO_3_) (Figure 4). The same trend was also observed under both the phosphate-excess and phosphate-limited culture conditions. The benefits of these findings are as follows. On one hand, the remaining nutrient of nitrogen or phosphate in the medium can be further utilized in the next cycle of semi-continuous cultivation. On the other hand, the EPS of *A. platensis* are high-value natural byproducts that could be applied on the cosmetic, food or pharmaceutical sector. It is a cost-effective culture method to obtain high-value biologically active substances by adjusting the cheap nutrient of the culture medium.

## 4. Conclusions

Recycling of the culture medium significantly (*p <* 0.01) increased EPS yield but reduced the biomass production of *A. platensis.* The medium with initial pH of 11.5 had the strongest inhibitory effect on *A. platensis*, mainly reflected in biomass production (0.38 ± 0.14 g/L), the lowest growth rate (0.10 ± 0.02 day^−1^) and the highest EPS yield (92.87 mg/L), respectively. The limitation of nitrogen (<2.50 g/L) and phosphate (<0.50 g/L) reduced the biomass production of *A. platensis*. As a result of salt stress, the EPS secretion and growth inhibition of *A. platensis* were significantly (*p <* 0.01) increased when nitrogen (25.00 g/L) and phosphate (>5.00 g/L) were excessive. The findings of the study not only provided technical guidance for the future two-step culture method for high-yield EPS without affecting biomass production, but also testified the potential of realizing low-cost and high-efficiency EPS production by regulating culture conditions.

## Figures and Tables

**Table 1:** Experimental set-up of the cultures using nitrate (NaNO_3_) and ammonium (NH_4_Cl) as nitrogen source.

Treatment	NaNO_3_ (g/L)	NH_4_Cl (g/L)	N Concentration (N g/L)
1	0.00	-	0.00
2	0.50	-	0.08
3	2.50	-	0.41
4	5.00	-	0.82
5	25.00	-	4.12
6	-	0.32	0.08
7	-	1.57	0.41
8	-	3.16	0.82
9	-	15.74	4.12

## Data Availability

The data presented in this study are available on request from the corresponding author. The data are not publicly available due to privacy.
